# Narrowed Meatus as a Cause of Stricture of the Urethra

**Published:** 1877-01

**Authors:** John Thad. Johnson

**Affiliations:** Professor of Anatomy and Lecturer on Venereal Diseases in Atlanta Medical College. Atlanta


ATLANTA

MEDICAL AND SURGICAL JOURNAL

VOL. XIV.] JANUARY—1877. [No. 10

### Original Communications.

### NARROWED MEATUS AS A CAUSE OF STRICTURE OF THE URETHRA.

BY JOHN THAD. JOHNSON, M.D.,
Professor of Anatomy and Lecturer on Venereal Diseases in Atlanta Medical College.

Below I present a paragraph briefly detailing two cases which I read before the Atlanta Academy of Medicine some months since. By an oversight on my own part, it was not embodied in the reporter's abstract. I present it here for the purpose of presenting additional cases, and making a few comments on the affection. The paragraph alluded to follows:

Two cases recently came under my treatment that presented some points of interest. The patients were young men—brothers. In each there were two strictures of the urethra—one in the spongy, the other in the membranous part. Each had, also, a constricted meatus, congenital. Neither had ever had any venereal disease, nor any injury of the urethra. The meatus in one scarcely admitted a filiform bougie. The strictures in this patient were more extensive and of smaller calibre than in the other—admitting only the filiform bougie. The strictures were not spasmodic, as a result of the abnormal meatus, but were organic. I believe, however, that the constricted meatus was the *cause* of the deeper strictures—these being developed by the chronic irritation which the meatus produced, just as chronic gonorrhœa produces stricture. This I regard as the most interesting point in the cases. Internal urethrotomy was performed on each.

Since the above was written I have operated on at least three

ATLANTA

MEDICAL AND SURGICAL JOURNAL.

other strictures caused by a congenital constricted meatus. They did not occur in the same family, as in the two above given. My impression is that I had treated other cases before the report to the Academy, in which there was no cause but the meatus to produce the stricture, but as the condition had not then particularly attracted my attention, I made no special investigation of them.

Heretofore, gonorrhœa or traumatic injury have been looked upon as almost the only cause of strictured urethra. The object of this paper is to call attention to the small meatus as a by no means infrequent cause. The meatus need not be nearly so small as in the cases first related above. A meatus, in a penis of average size, that will not admit an English No. 12 (French 23) without painful stretching, is not of normal size. I do not claim, however, that a meatus so large as this will necessarily, or even probably, produce a tight stricture. But one of even this size may produce a lesion sufficient to set up disorders both of the seminal and urinary function. A meatus as small as No. 7 or 8, English, will almost inevitably produce such lesions. Symptoms may be present in early life; they more constantly appear after the age of puberty.

The constricted meatus, even when it has been productive of no lesion or disordered function, may prove a serious inconvenience if the patient should contract a gonorrhœa, rendering the disease difficult of cure, prolonging it indefinitely, thereby producing a stricture of the canal.

This abnormal meatus may possibly set up, especially in early life, an inflammation of the urethra. In such case we may charge the boy wrongly, or be ourselves at a loss to account for his disease.

We can well see how this condition may produce incontinence of urine in boys. It forms a stricture deeper in the canal, and there results an irritability at the neck of the bladder, producing too ready a discharge of the contents of the bladder—a symptom which, in adults, is very familiar to surgeons as a consequence of stricture. Many boys have been dosed with belladonna, syrup of iodide of iron, and countless othes remedies, when the trouble could have been more effectually relieved by a stroke of the knife.

We meet with disorders of the seminal functions as a consequence of stricture, the result of a urethritis, in the adult. We also may find such trouble very early after puberty as a symptom of small meatus and its attendant stricture.

As already intimated, the seminal and urinary disorders attending a narrow meatus, or stricture, depend directly upon the irritability or *hyperesthesia* of the neck of the bladder. In a recent discussion in the Atlanta Academy of Medicine, as reported in the December number of the JOURNAL, I state that such disorders depend upon hyperesthesia of the *glans penis.* I take this opportunity of correcting the statement. I intended to say that they depend on hyperesthesia of the *neck of the bladder.*

The stricture resulting from the small meatus is not merely spasmodic, but a true organic lesion. This I have demonstrated by careful observation of the stricture after division of the meatus.

The narrow meatus, if very small, I believe will almost inevitably produce the deeper lesion, unless relieved in early life. The stricture may become as small in calibre as though produced by an ordinary urethritis.

There is but one treatment for constricted meatus—division with the knife, or instrument specially devised for such operation. The deeper lesion should be treated as if produced in the more usual way.

I might elaborate some of the above-given points to considerable length, but my present object is to hurriedly call attention to the congenitally narrow meatus as a cause of stricture and its consequent symptoms.

